# Development of an Ultra-Sensitive and Flexible Piezoresistive Flow Sensor Using Vertical Graphene Nanosheets

**DOI:** 10.1007/s40820-020-00446-w

**Published:** 2020-05-11

**Authors:** Sajad Abolpour Moshizi, Shohreh Azadi, Andrew Belford, Amir Razmjou, Shuying Wu, Zhao Jun Han, Mohsen Asadnia

**Affiliations:** 1 School of Engineering, Macquarie University, Sydney, NSW 2109 Australia; 2 UNESCO Centre for Membrane Science and Technology, School of Chemical Science and Engineering, University of New South Wales, Sydney, NSW 2052 Australia; 3 CSIRO Manufacturing, PO Box 218, 36 Bradfield Road, Lindfield, NSW 2070 Australia

**Keywords:** Vertical graphene nanosheets, Artificial vestibular system, Bioinspired sensors, Piezoresistive sensors

## Abstract

This paper suggests development of a flexible, lightweight, and ultra-sensitive piezoresistive flow sensor based on vertical graphene nanosheets (VGNs) with a mazelike structure. The sensor was thoroughly characterized for steady-state and oscillatory water flow monitoring applications. The results demonstrated a high sensitivity (103.91 mV (mm/s)^−1^) and a very low-velocity detection threshold (1.127 mm s^−1^) in steady-state flow monitoring. As one of many potential applications, we demonstrated that the proposed VGNs/PDMS flow sensor can closely mimic the vestibular hair cell sensors housed inside the semicircular canals (SCCs). As a proof of concept, magnetic resonance imaging of the human inner ear was conducted to measure the dimensions of the SCCs and to develop a 3D printed lateral semicircular canal (LSCC). The sensor was embedded into the artificial LSCC and tested for various physiological movements. The obtained results indicate that the flow sensor is able to distinguish minute changes in the rotational axis physical geometry, frequency, and amplitude. The success of this study paves the way for extending this technology not only to vestibular organ prosthesis but also to other applications such as blood/urine flow monitoring, intravenous therapy (IV), water leakage monitoring, and unmanned underwater robots through incorporation of the appropriate packaging of devices.

**Electronic supplementary material:**

The online version of this article (10.1007/s40820-020-00446-w) contains supplementary material, which is available to authorized users.

## Highlights

- A novel biomimetic flow sensor based on vertically grown graphene nanosheets with a mazelike structure is fabricated which closely mimics the structure of auditory hair cells.
- The proposed sensor demonstrated an ultra-high sensitivity of 103.91 mV (mm/s)^−1^, very low-velocity detection threshold (1.127 mm s^−1^), and excellent performance in a wide range of frequencies (0.1–25 Hz) for underwater sensing applications.
- The proposed sensor revealed a strong capability in development of an artificial lateral semicircular canal.

**Electronic supplementary material:**

The online version of this article (10.1007/s40820-020-00446-w) contains supplementary material, which is available to authorized users.

## Graphical abstract

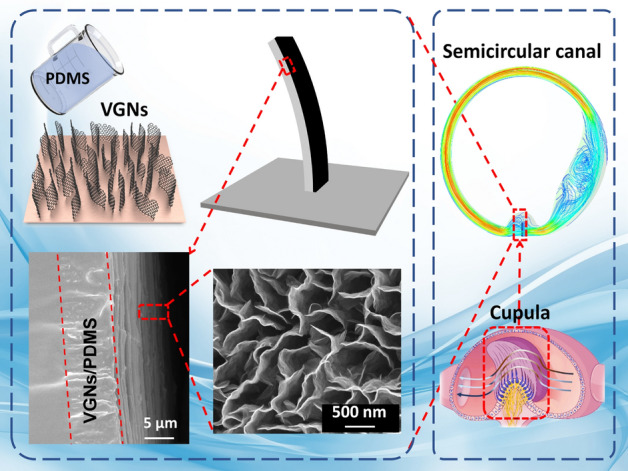

## Introduction

Biological sensors (biosensors) exist in all creatures capable of filtering, measuring, and recording biologically relevant signals in a noisy environment. A biosensor serves as an analytical device converting a biological response into a measurable signal [1, 2]. High sensitivity, wide dynamic range and mechanical robustness are valuable characteristics of biological sensors [3]. Through billions of years, natural biosensors including flow sensors, acoustic sensors, and chemical sensors have evolved, developing extreme efficiency, compactness, high responsivity, and throughput [4]. Among all the biosensors, hair cell (cilia), as multipurpose mechanical transducers, is ubiquitous in a vast range of living beings, including amphibians, mammals, insects and fish. They react quickly to flow, vibration, touch, acoustic vibration and gravitational force [3]. Sensing via the cilia is based on mechanical displacement induced by flow, generating electrochemical signals and sent to the brain. These sensory hair cells include the lateral line system of fishes [5, 6], the vestibular system of birds and mammals [7], joints in insects for flow detection [8], and amphibians for flow and vibration sensing [4].

Many researchers have devoted considerable effort to mimicking of biological hair cell sensors, particularly the fish lateral line system. Water flow rate and direction can be accurately detected using these sensory hair cells. Such sensors can be mainly classified based on sensing method into piezoresistive effect [9, 10], piezoelectric effect [11, 12], capacitive principle [13–16], and ionic polymer–metal composites (IPMC) [17–20]. Two main factors that play a pivotal role in efficient flow biosensors are high sensitivity and resolution. Recently, many research efforts have been undertaken in order to fabricate a useful flow sensor that can mimic sensory hair cells. Kottapalli et al. [21] developed a MEMS hair cell sensor using a polycarbonate hair cell structure mounted at the center of a patterned liquid crystal polymer (LCP) membrane. This sensor measures the steady-state flow velocities with threshold velocity detection limits of 0.1 m s^−1^ and oscillatory flows. For interested readers, comprehensive review papers on MEMS flow sensors [22] and artificial hair cell sensors [23] have recently been published.

In this work, we focused on designing a novel sensory hair cell inside three interconnected semicircular canals (SCCs) located in the vestibular system. SCCs are one of the main parts of the vestibular system and are fundamental to normal movement and balance of the body. Specifically, the SCCs sense the angular movements of the head, while the other parts of the vestibular system, the utricle and the saccule, detect gravitational and inertial movements. Each SCC comprises a slender duct, the utricle, and ampulla. The utricle is shared by the three SCCs. The SCCs are filled with a biological fluid called endolymph, whose mechanical properties are quite similar to water [24]. Sensory hair bundles positioned in the ampulla are deformed by the movement of endolymphatic flow induced by head movement. Sensory system as mechanoreceptors cells inside the SCCs is about 70 μm in height, 44 nN m^−1^ compliant to bending, and has a 10 μm tip displacement threshold [25]. A schematic image of the biological semicircular canal is shown in Fig. S1. Many numerical investigations [26–28] have been conducted to analyze the hair cell deformation over oscillatory movements; however, few studies on the vestibular system have been experimentally undertaken. Most of the experiments during the last two decades used accelerometers and gyroscopes [29–31] for detecting linear motion and rotational motion, respectively. Making a suitable flow sensor by taking inspiration from nature in mimicking sensory hair cells inside SCCs has been fast gaining attention among researchers in recent years. Sharif and Tan [32] fabricated a pressure gradient sensor using IPMC-embedded cupula structure positioned into a U-shaped channel. Their work only studied the pressure difference induced by dipole vibration. Our group previously introduced a MEMS flow sensor for use in a three-dimensional (3D) printed SCC at a wide range of frequencies and rotational angles around three rotational axes, namely yaw, pitch and roll [33]. The flow sensor was fabricated using a membrane-based pressure sensor inspired by cavefish lateral line hair cell sensor.

Graphene has been recognized as a promising sensing material for mimicking biosensors, due to its remarkable structural and electrical properties. Graphene consists of two-dimensional analog of fullerenes and carbon nanotubes with one-atom thickness in a hexagonal lattice [34]. Due to its unusual structure, graphene and its by-products demonstrate numerous remarkable characteristics including high planar surface, great elasticity, tunable optical properties, and high mechanical strength [35, 36]. In addition, graphene possesses a high signal-to-noise ratio, allowing detection at room temperature of small electrical perturbations [37], making it attractive for a wide range of sensing applications, including electrochemical biosensors [38–45], flow sensors [46, 47], stretchable strain sensors [48, 49] and gas sensors [50–52]. The outstanding feature of graphene is its piezoresistive effect, causing the resistivity of graphene to vary linearly with applied strain [53, 54]. However, because of a bandgap not opening in graphene under uniaxial strains, it is challenging to use this material for the applications that require high piezoresistive sensitivity [55]. For instance, Kamat et al. [56] recently developed a flexible flow sensor with a cantilever-based structure with micropillar and a flexible strain gauge, made from a solution of ethanol and graphene nanoplatelets. Under cyclical tension–compression tests, the graphene solution strain gauge showed a gauge factor of 37 for strain ± 1.92. To improve sensitivity, many research efforts have been made with the aim of optimizing the structure of graphene. For the first time, Li et al. [57] made graphene-based woven fabric (GWF) by intersecting graphene micron-ribbons (GMRs). This structure has notable advantages over planar network structures, such as high permeability and better mechanical performance. Later, the ultra-high sensitivity of graphene was achieved by using macrowoven fabric structure with a gauge factor of 500 for strains below 2% [58]. Compared to a planar network structure of patterned graphene, the graphene woven fabric produces a large interfacial resistance between the opened gaps and the interlaced ribbons, thereby improving sensitivity [59]. However, simultaneously improving stretchability or bendability and sensitivity in flow sensors has been a significant challenge.

In this work, for the very first time, we fabricated a miniaturized flow sensor based on vertically grown graphene nanosheets (VGNs) with penetrated PDMS and a mazelike network, specifically for the applications of oscillatory and rotational flows. VGNs are two-dimensional graphene nanosheets oriented perpendicularly on a substrate produced by a plasma-enhanced chemical vapor deposition (PECVD) process [60]. Vertically oriented graphene nanosheets have many advantages over the majority of horizontally grown graphene-based sensors due to an open, interwoven 3D structure porous to fast transport analyte molecules, and a mechanically rigid structure able to prevent the graphene from restacking [59, 60]. The proposed flow sensor consists of VGNs as the sensing component with PDMS cast into open pores of the VGNs to form a flexible piezoresistive sensor. The VGNs/PDMS sensors with high conductivity exhibit distinctive capabilities of linearity, low threshold velocity, ultra-high sensitivity and ultra-lightweight. To demonstrate these features of the flow sensor, we conducted experiments to study the sensor response to a harmonically moving sphere in air and water, and to steady-state flow of water in a straight channel. Finally, to demonstrate its application, the sensor was embedded into a biomimetic semicircular canal and mounted on a rotary table able to mimic human head movement. In addition, the effectiveness of the flow sensor was assessed by substituting a circular canal for the semicircular canal, then comparing results.

## Experimental Section

### Fabrication of VGNs and VGNs/PDMS Flow Sensors

**Fig. 1:**
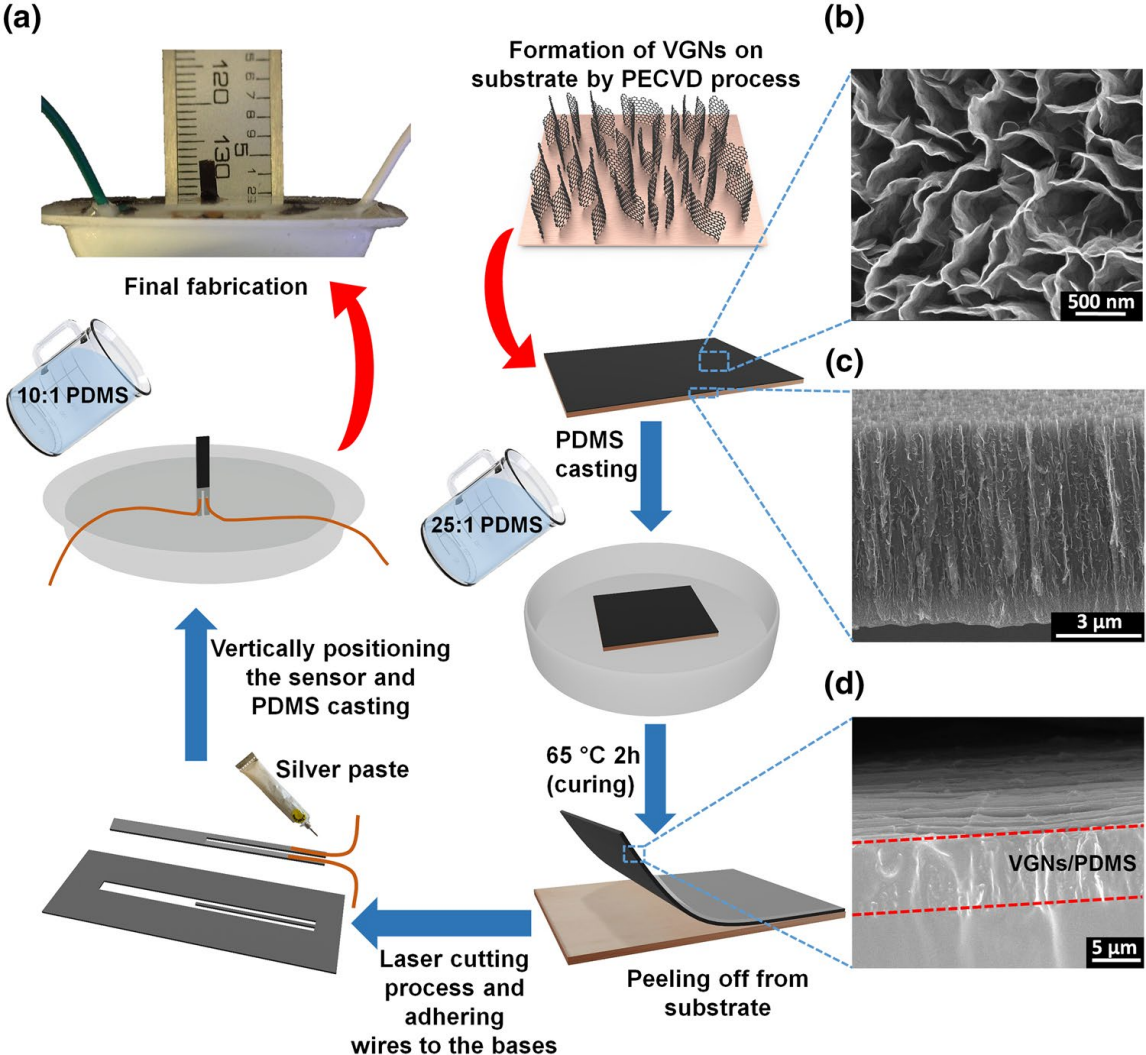
**a** Schematic diagram of the fabrication process and SEM micrographs of VGNs and VGNs/PDMS. **b** Mazelike structure of vertical graphene nanosheets. **c** Side view of the vertically grown graphene nanosheet layer. **d** Side view of sensing element including vertical graphene nanosheet coated with PDMS layer

The detailed process of synthesis and growth mechanism of VGNs using PECVD technique, and complete characterization has been described elsewhere [59, 60]. For this work, VGNs were fabricated with a height of 7 μm. Removing the VGNs from the substrate while conserving their properties and morphology is a challenging process due to their delicate nature [61]. We adopted a new approach to overcome this issue, infiltrating the VGNs with PDMS, allowing the VGNs along with cured PDMS to peel off from the substrate easily. The fabrication process is illustrated in Fig. 1. PDMS prepolymer and curing agent (Sylgard 184, Dow Corning Australia) in several mixtures, 10:1, 15:1, 20:1, 25:1, and 30:1 weight ratios were prepared by magnetically stirring the liquid PDMS solution for 15 min and degassing it for additional 25 min, which facilitates the infiltration of liquid PDMS into the porous structure of the VGNs. After curing at 65 °C for 2 h, the thin film of VGNs/PDMS was peeled off from the copper foil. Then, the mechanical properties and rheological properties of all mixtures were measured using a universal testing machine (MTS Exceed E42, MTS Systems Corporation, USA) and a stress-controlled shear rheometer (MCR 302, Anton Paar, Graz, Austria). The results obtained from these tests are presented in Figs. S2 and S3. By comparing the results for stiffness, the weight ratio of 25:1 for PDMS prepolymer and curing agent was chosen in order to have high sensitivity to a minimal stimulus. To shape the flow sensor, a rectangular parallelepiped shape with 1 mm width, 6.5 mm height, and 0.5 mm thickness was cut from the VGNs/PDMS thin film by a laser cutter apparatus in such a way that two separate legs were created. By adhering two copper wires onto the two ends of the surface of VGNs by silver paste, the flow sensor was prepared for electromechanical tests. In order to keep the sensor vertical, the flow sensor was embedded into a PDMS base (Fig. 1). The final height of VGN standing structure is 4.5 mm.

### Characterization of the VGNs/PDMS Flow Sensors

#### Structure of VGNs/PDMS Flow Sensor

The morphologies of the VGNs and its PDMS nanocomposites were revealed by scanning electron microscopy (SEM) characterization. The samples were coated with a thin platinum layer prior to scanning. In addition, they were immersed in liquid nitrogen and cross sectionally broken in order to characterize the cross-sectional microstructure.

#### Sensing Performance Characterization

**Fig. 2:**
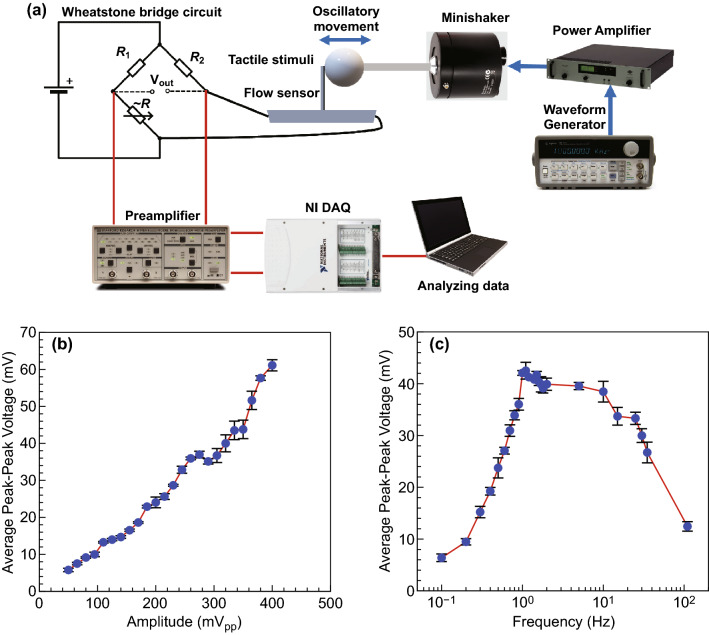
**a** Schematic diagram of the experimental setup for oscillatory stimuli in contact with the sensor (in air). **b** Average peak-to-peak voltage as a function of amplitude at *f* = 10 Hz. **c** Average peak-to-peak voltage as a function of frequency at amplitude of 354 mV_pp_

The performance of the sensor in response to dynamic flows was measured using an oscillatory sphere (dipole) which vibrates in the vicinity of the sensor. Fresh deionized (DI) water was used for all experiments to ensure the base resistance of the sensor remains unchanged. The experimental setup for dipole in air and water is shown in Fig. 2a. A sphere (16 mm in diameter) and a rod (2 mm in diameter and 100 mm in length) constitute the dipole. The 3D printed dipole was securely screwed onto a permanent magnet mini-shaker (model 4810, B&K, Norcross, GA, USA). A waveform generator (Agilent 33120A Function/Arbitrary) connected the mini-shaker was used to drive the dipole at the desired amplitudes and frequencies. The generator was attached to the power amplifier (type 2718, B&K) to amplify the signals with a specific gain (10 dB). The dipole induced deflection of the sensor results in the changes in the resistance of the VGN, which is transformed into the voltage through a Wheatstone bridge circuit with resistance values *R*_1_= 9.98 kΩ, *R*_2_= 9.96 kΩ, the resistance of potentiometer, ~ *R* = 6.75 kΩ, and the supply voltage, *V*_in_= 6.36 V (Fig. 2a). The voltage signals were filtered using an SRS560 low-noise preamplifier with unity gain; then, the filtered voltage was measured by a National Instruments (NI) NI-9239 Data Acquisition (DAQ) device. Finally, NI LabVIEW Signal Express software was used to analyze the data. To ensure the reproducibility of the results, each experiment was repeated three times using three different sensors (nine separate tests). Error bars show the standard error for several repeated tests for the same setup. The fixed values of frequency and amplitude were considered to be 10 Hz and 354 mV_pp_ (peak-to-peak voltage), respectively. The amplitude of vibration of the dipole can be translated to approximate flow velocity or distance as described in the previous study using a laser Doppler vibrometer (LDV) [62]. For the dipole experiments, the flow sensor and dipole were immersed in a water tank of dimensions 25 × 45 × 27 cm^3^, which was filled with DI water to a height of 23 cm.

Steady-state flow testing is an essential procedure to characterize the flow sensor. For this purpose, the flow sensor was calibrated under steady-state DI water flow inside a straight channel designed with SOLIDWORKS software and 3D printed with Clear resin by an SLA printer. (The experimental setup for steady-state flow testing is shown in Fig. 4a.) A straight channel was chosen for generating a fully developed fluid flow across the channel. The channel size is 8.6 × 8.6 × 120 mm^3^, with a hole that was provided for the sensor to be embedded 70 mm downstream from the entry port. The channel inlet was connected to a plastic syringe by a hose with a diameter of 1.5 mm. The fluid flow experiences an abrupt expansion at the inlet of the channel, so the position of the flow sensor was set at a sufficient distance from the entrance, based on the formulation of entrance length for abrupt expansion, *L*_e_= 0.044*D*_*h*_*Re* [63]. This ensured that the sensor was positioned in the fully developed flow region. The syringe was equipped with a syringe pump to apply a constant steady-state flow condition. To convert the resistance changes originating from the deflection of the VGNs thin film into voltage, the sensor was connected to a Wheatstone bridge circuit, and the resultant voltage was directly measured through a DAQ device. In addition, a voltage divider circuit was used to collect the sensor output in terms of resistance change. The data obtained were processed with NI LabVIEW Signal Express software to determine the sensor output voltage. This experiment was conducted for the flow velocities in a range of 5–37 mL min^−1^. Setting the pump on a specific velocity and energizing it caused the syringe to inject water into the channel, creating a fluid flow over the sensor. The flow deforms the VGNs thin film, varying the electrical resistance.

## Results and Discussion

### Structure of VGNs/PDMS Flow Sensor

The morphology of VGNs and the VGNs/PDMS thin film flow sensor was examined by SEM, and Fig. 1b–d shows the corresponding SEM images. Figure 1b (top view of the VGNs) indicates that several layers of graphene nanosheets constitute the VGN walls, whose thickness was measured to be roughly 1–5 nm. The VGNs height was estimated to be around 7 μm, as displayed in Fig. 1c (cross-sectional view of VNGs). From this figure, there are also some graphene nanosheets perpendicular to the VGNs walls. These branches, along with walls, generate open cavities with maximum sizes of the order of 1 µm (Fig. 1b). Therefore, polymers such as PDMS can infuse these open pores, thereby improving the stretchability and flexibility of the VGNs/PDMS thin films. As shown in Fig. 1d, the thin film has two layers with the top being VGNs/PDMS and the bottom being pure PDMS layer. The thin film shows high electrical conductivity due to the existence of a mazelike network of VGNs walls.

### VGNs/PDMS Thin Film as a Flow Sensor

In this work, we explore the application of VGNs/PDMS thin film as an ultra-sensitive and flexible miniaturized flow sensor. Moreover, the proposed sensor has been thoroughly characterized in both oscillatory and steady-state flow.

#### Piezoresistivity of VGNs/PDMS in Response to Tactile Oscillatory Stimuli

One critical characterization of a flow sensor is to monitor its response to oscillatory stimuli applied by dipole vibrations. The setup is shown in Fig. 2a, and the responses of the VGNs/PDMS thin film to oscillatory stimuli (amplitude sweep and frequency sweep) are given in Fig. 2b, c.

In addition to flow sensing, hair cell sensors in nature detect vibrations and touch stimuli, so the performance of the sensor was measured under oscillatory stimuli in direct contact with the sensor in which oscillatory frequency and amplitude were precisely controlled, as shown in the schematic diagram of the experimental setup (Fig. 2a). The response of the VGN/PDMS flow sensor to oscillatory contact stimulation was studied by the variations in frequency and amplitude of vibrations over a wide range (0.1–110 Hz and 50–400 mV_pp_, respectively), as shown in Fig. 2b, c. From Fig. 2b, the flow sensor follows a roughly linear upward trend with amplitude. Increasing amplitude causes the sensor to bend further, meaning that an increase in bending angle over the sensor generates a higher strain on the VGNs, thereby changing the electrical resistance. The piezoresistivity of VGNs/PDMS is governed by three main mechanisms, as reported in Ref. [48]. During the stretching cycle that the VGNs/PDMS nanocomposite experiences, the conductive network formed by VGNs is altered, leading to an increase in contact resistance and tunneling resistance. The graphene nanosheets are also deformed. Therefore, the intrinsic piezoresistivity of graphene may also contribute to the overall piezoresistivity of the VGNs/PDMS. Upon release of the load, the nanocomposite recovers to its state prior to deformation, resulting in the recovery of the resistance. The data extracted from the frequency sweep can be divided into two regions, as shown in Fig. 2c. An upward trend in sensor output originating from the increase in frequency is seen in the lower frequency range (0.1–1.1 Hz), while a decreasing trend is observed after 1.1 Hz. Increasing the frequency in oscillatory stimuli causes an increase in strain rate due to stretching and releasing the VGNs thin film. Actually, stretching induces deformation in the VGNs network, while during release, the conductive network contracts and recovers [64, 65]. At high frequency, this recovery may not be completed due to the inherent viscoelastic nature of the elastomer (PDMS) matrix and overshooting phenomenon, and consequently, the resistance of the VGNs thin film drops.

#### Piezoresistivity of VGNs/PDMS in Response to Oscillatory Stimuli in Water

**Fig. 3:**
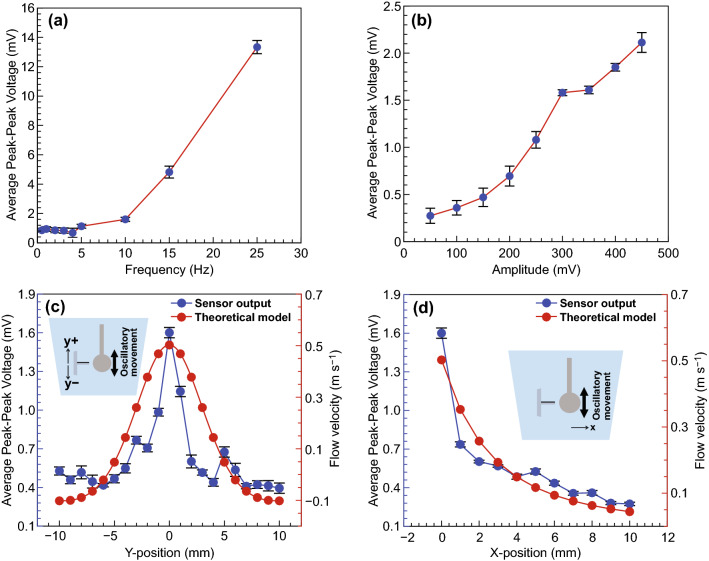
**a** Average peak-to-peak voltage as a function of frequency for oscillatory flow characterization and object detection principles. **b** Average peak-to-peak voltage as a function of amplitude. **c** Position sweep in the *y*-axis direction (parallel). **d** Position sweep in the *x*-axis direction (perpendicular)

The main goal of the VGN/PDMS flow sensor is to work under conditions of oscillatory flow, so it is critical to observe the sensor response when subjected to periodic flow variations. The dipole was located near the sensor at the origin of the *y*-axis and very close to the origin of the *x*-axis (the sensor tip) for studying the frequency and amplitude variation. The vibration of the dipole near the sensor leads to movement of the water, generating a wave which deflects the sensor, changing its electrical resistance. The water movement due to gravity, rotational motion, and temperature differences was deemed to be negligible. Increasing the frequency of the dipole movement increases the wave amplitudes, leading to larger deformation in VGNs thin film. As shown in Fig. 3a, an upward trend is observable in amplitude sweep test with a linear behavior, while there is an exponential trend in average peak–peak sensor output with the increase in frequency as in Fig. 3b. It is worth noting that the signal that the sensor reads by indirect application in water under oscillatory movement is significantly different to that from direct contact with the dipole, because the flow sensor senses the forces from the waves in water, rather than directly being bent by the dipole.

The device’s ability to determine the distance of the dipole from the sensor is experimentally investigated. For these experiments, frequency and amplitude of vibration were kept constant at values of 10.0 Hz and 354 mV_pp_, respectively, while the position of the dipole with respect to the sensor was varied parallel (*y*-axis) and perpendicular (*x*-axis) to the base of the sensor. Initially, the dipole is located at the center of the sensor and very close to the standing structure without touching it.

Figure 3c shows the sensor output when the position of the dipole along the *y*-axis changes with intervals of 1 mm, while its position with respect to sensor in the *x*-axis maintained constant. In total, 20 points were collected as can be seen in Fig. 3c. Through this experiment, it is possible to compare the sensor’s behavior when the flow is toward the VGNs thin film side (positive *y*-axis) against with the case that the flow is toward the PDMS side (negative *y*-axis).

The maximum output of the sensor (1.6 mV) was recorded when the dipole was placed at close vicinity of the sensor. Ten observation distances corresponded to the negative positions, while another ten were related to the positive positions. The overall response shows a clear peak around the initial position of the vertical movement of the sensor with a gradual decrease in the response as the distance increases, resembling a “Mexican hat profile” response. This is due to the displacement of water particles caused by the vibration of the dipole, leading to wave generation.

Moreover, the velocity of fluid flow generated by the vibrating dipole can be theoretically defined as Eq. (1) [62],

1 $$V = a\omega A^{3} \frac{{\left( {2y^{2} - P\left( x \right)^{2} } \right)}}{{\left( {y^{2} + P\left( x \right)^{2} } \right)^{2.5} }}$$

where *A* is the sphere diameter (*A* = 16 mm), *P*(*x*) is the distance between the center of the sphere and the sensor tip, *a* is the sphere displacement amplitude (estimated *a *= 1 mm), and *ω* is the angular vibration frequency (*ω* = 2*πf*, *f* = 10 Hz). The results presented in Fig. 3c demonstrate that as predicted by the theoretical analysis, the spatial response of the sensor resembles a “Mexican hat” profile, which is in agreement with previous studies on neuro-physiological of hair cell sensors [66].

A further experiment was conducted to investigate the sensor response to the dipole vibration at different distances in the x-direction, as shown in Fig. 3d. The dipole was located in 11 positions with a constant increment, from very close to the sensor tip (almost zero distance) to 10-mm distance. The highest measured output of the sensor was 1.6 mV when the dipole was very closed to the sensor. This value reduced as the dipole positioned further from the sensor to a minimum of 0.27 mV when the dipole reached 10 mm away from the sensor. As expected, the sensor output reduces with increasing distance between the vibrating dipole and flow sensor because of the decrease in the power of the generated pulses. For simulation of the flow velocity produced by the dipole vibration in *x*-direction, Eq. (1) is used by substituting *y* = 0 and *x* varying from 0 to 10 mm. (*P*(*x*) varies from 8 to 18 mm.) Figure 3d compares the sensor response to varying the dipole position related to the sensor with the flow velocity generated by the vibrating dipole at different positions.

#### Piezoresistivity of VGNs/PDMS in Response to Steady-State Flow

**Fig. 4:**
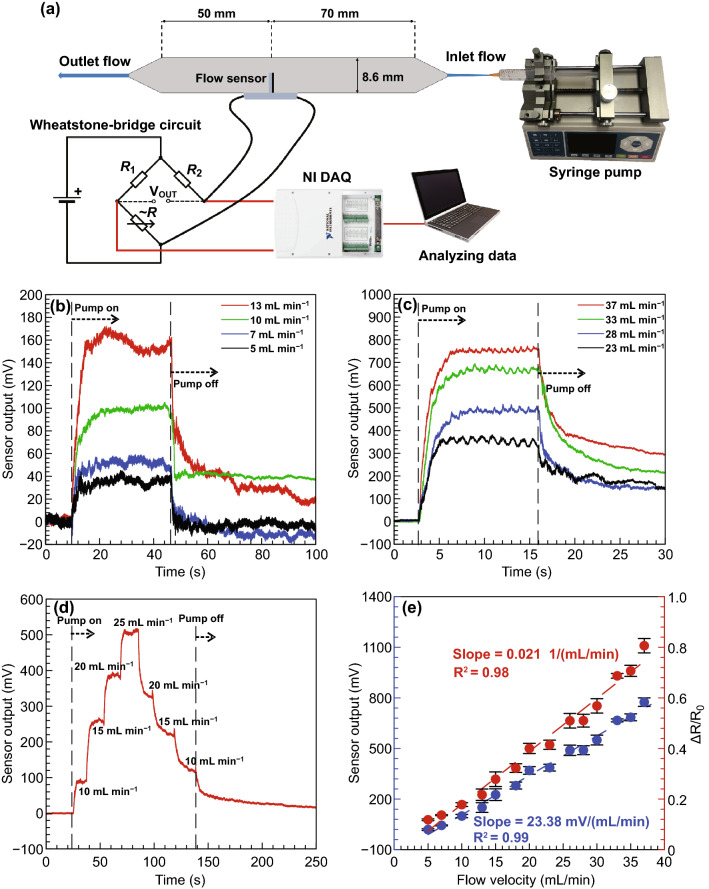
**a** Schematic diagram of the experimental setup for steady-state flow testing inside a straight channel. **b** Sensor output for low flow velocities and **c** high flow velocities. **d** Sensor output as a function of time for various flow velocities. **e** Sensor output as a function of flow velocity (calibration plot)

To better show the effect of various flow velocities on the sensor output, the results of low and high velocities are demonstrated in two separate figures (Fig. 4b, c). Before applying the flow to the sensor, the bias stability of the device was measured (Fig. S5). The resistance deviation from the baseline (6.08 kΩ) was around 10 Ω, which is negligible considering the sensitivity of the sensor, which is 127 Ω (mL/min)^−1^, as presented in Fig S7. By applying a constant flow velocity via the syringe pump, the VGNs thin film begins to be bent due to force from the fluid flow. After the flow reaches a determined velocity and settles, the thin film reaches the maximum bend measurement. Under bending, the deformation of the VGNs conductive network causes its resistance to increase. Therefore, once the pump is turned on, the sensor output voltage surges from its initial value to the maximum when the flow reaches a steady-state condition; following that, the sensor output plateaus. As shown in Fig. 4b, c, the response time (steady-state response) is about 5 s for low velocities and 3 s for high velocities. When the pump is turned off, the VGNs thin film is being released, so the deformation recovers and the sensor output plunges sharply. However, the final resistance of the sensor is slightly different from the initial value and takes some time to reach the initial base due to the inherent viscoelastic nature of the PDMS matrix and small deformation in graphene networks (overshooting phenomenon) [48, 59, 64]. Overshooting occurs when a small deformation in layers of polymer leads to change the distance between several nanomaterials and thereby varying the total resistance of the sensor. At low velocities, the VGNs thin film has enough time to adapt to the fluid flow and the slight deformation in the nanostructure of polymer layers recovers quickly. After the flow is stopped, the changes in the nanostructure return to the initial positions, so the resistance of the sensor reaches its baseline. At low velocities, the recovery time is far lower than that of high velocities as shown in Fig. 4b, c. For instance, for the flow rates of 5 and 7 mL min^−1^, the sensor output returns entirely to the initial value in around 3 s. The recovery time for the high flow velocity 30 mL min^−1^ is around 1300 s (21 min), as presented in Fig. S6.

A separate set of experiments was designed to examine the VGNs/PDMS flow sensor under increasing and decreasing flow velocities, as presented in Fig. 4d. Four flow rates from 10 to 25 mL min^−1^ at a constant interval of 5 mL min^−1^ were applied to the sensor, and the sensor output for each case was recorded. The sensor output behavior with decreasing velocities was also observed and is presented in Fig. 4d. These results demonstrate that the sensor is highly responsive to flow velocity step changes. In addition, the sensor output values at each flow velocity remain much the same, whether under an increasing or decreasing trend of flow velocity.

The final experiment was to calibrate the flow sensor (Fig. 4e) by measuring the steady-state value of the sensor output under various flows. The flow velocity at the sensor tip is determined by calculation from the dimensions of the syringe and the channel. The minimum applied flow rate by the syringe pump that the sensor could detect was 5 mL min^−1^, corresponding to a linear fluid velocity of 1.127 mm s^−1^ over the sensor tip. The sensor output in terms of the voltage measured by the Wheatstone bridge circuit is reported in Fig. 4e. Also, the resistance changes of the sensor by applying the different flow velocities were measured using the voltage divider circuit (Fig. S4) and NI DAQ device. The slope of the graph is indicative of the flow sensor sensitivity calculated as 23.38 mV (mL/min)^−1^ or 103.91 mV (mm/s) ^−1^, or in terms of sensor resistance, 0.127 kΩ (mL/min)^−1^ or 0.564 kΩ (mm/s) ^−1^. Based on the recent review paper on flow sensors [22], in comparison with different flow sensors in various categories such as MEMS piezoelectric sensors, diaphragm MEMS piezoresistive sensors, cantilever MEMS piezoresistive sensors, Calorimetric MEMS thermal sensors, and hot-wire/film MEMS thermal sensors, it is clear from the measured sensitivity, linearity, and threshold velocity of the sensor that it is an important advance in sensing technology, as shown in Table 1.

**Table 1:** Sensor performance comparison in terms of threshold velocity and sensitivity

Sensing element material	Configuration	Threshold velocity (m s^−1^)	Sensitivity (mV (mm/s)^−1^)	References
PVDF–TrFE film	Piezoelectric	14.86	4.8 × 10^−5^	Jung et al. [73]
PVDF nanofiber tip links	Piezoelectric	8 × 10^−6^	0.3	Asadnia et al. [74]
Si strain gauge	piezoresistive	0.025	6.98	Chen et al. [75]
LCP sensing membrane, Au gauge strain	Piezoresistive	0.05	90.5 × 10^−3^	Kottapalli et al. [76]
VGN/PDMS nanocomposite	Piezoresistive	1.127 × 10^−3^	103.91	Current work

### Application of VGNs/PDMS Flow Sensor

The main motivation behind the fabrication of this flow sensor is to mimic the function of the sensory hair cells located in the human ampulla, as a part of a semicircular canal (SCC). These small hair cells play a vital role in stabilizing gaze during head movement, and for spatial orientation. With aging or due to accidents, vestibular sensitivity is likely to decrease due to loss of hair cells, resulting in symptoms such as diminished balance, a heightened risk of falling, and deterioration in dynamic visual acuity [67]. This is why taking inspiration from nature to mimic sensory hair cells is vital.

**Fig. 5:**
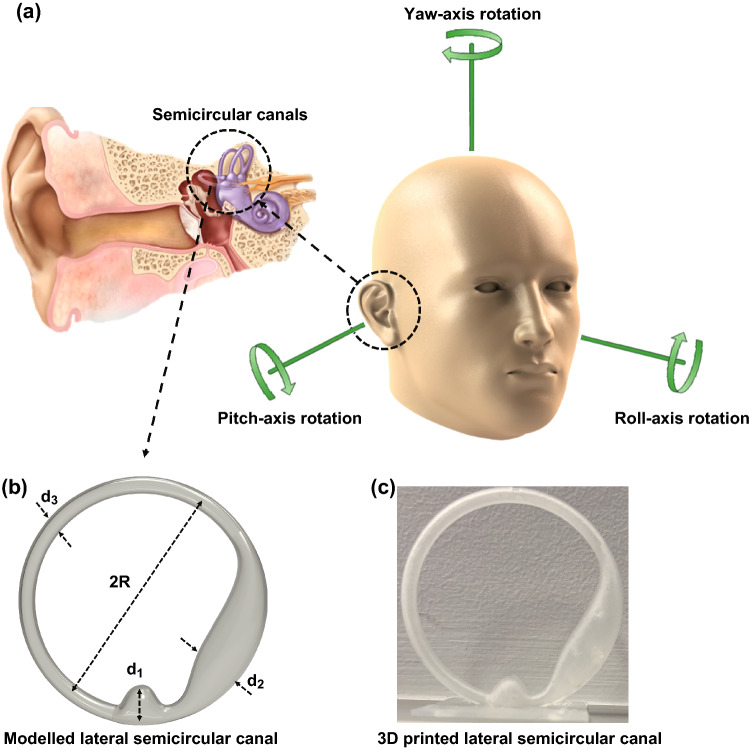
**a** Magnified view of the inner ear and the rotational axis for head movement. **b** Modeled LSCC. **c** 3D printed LSCC

The first step was to model the semicircular canal. The SCCs are composed of three interconnected ducts, namely posterior, anterior, and lateral (horizontal) SCCs. Each canal is orientated in a different plane that is approximately perpendicular to each other and maximally senses the rotation perpendicular to the canal plane. So the lateral semicircular canal (LSCC) is maximally sensitive to the yaw axis rotation, while the posterior and anterior canals maximally detect roll and pitch axes rotations [68, 69], respectively, as shown in Fig. 5a. For the sake of simplicity, we considered a straightforward model of the LSCC according to human morphological data [70] through which the geometry of LSCC can be characterized by four critical components including the diameters of the utricle (d_2_), slender duct (d_3_), and ampulla (d_1_), as well as the duct curvature (2R), as presented in Fig. 5b. To find the dimensions of these components, the inner ear of a human was investigated by a 3T magnetic resonance imaging (MRI) scanner (Siemens, Verio). The morphological data of the LSCC are presented in Fig. S7; however, for simplifying the fabrication process, dimensions were multiplied sixfold. The LSCC was developed in SOLIDWORKS software and printed by a Stereolithography apparatus (SLA) 3D printer with clear resin (Fig. 5c).

**Fig. 6:**
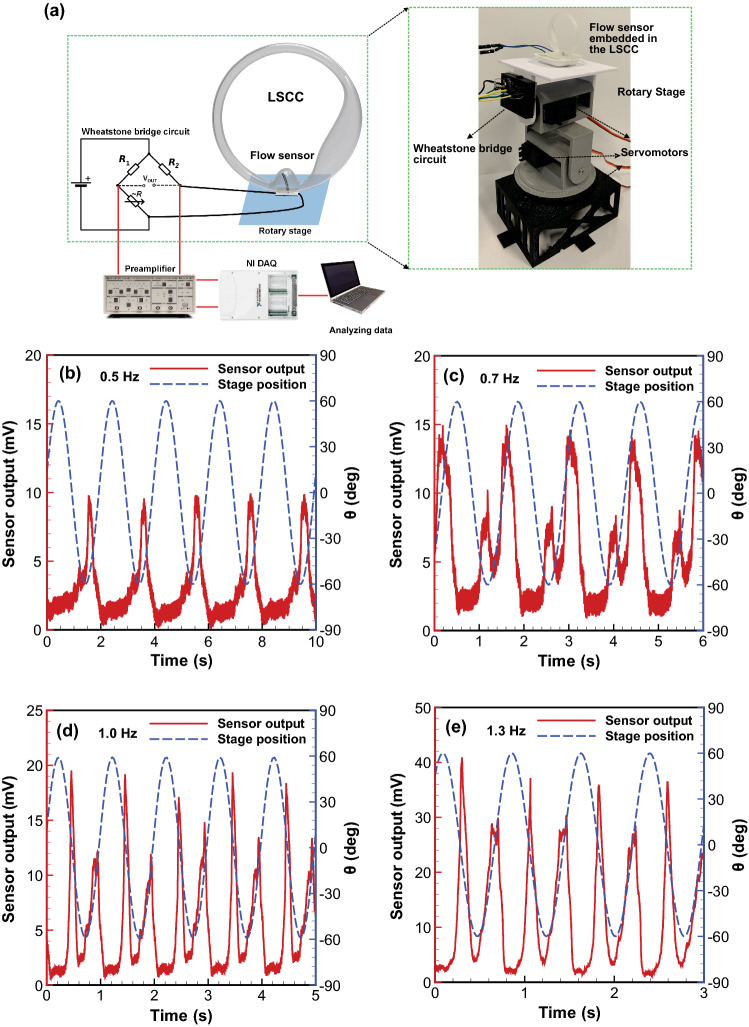
**a** Schematic diagram of the experimental setup, and sensor-stage position for 60-degree amplitude and various frequencies: **b** 0.5 Hz, **c** 0.7 Hz, **d** 1 Hz, and **e** 1.3 Hz

The next step was to simulate the movement of the human head. Head rotation in the yaw, pitch, and roll axes does not exceed angles of 80°, 40°, and 60°, respectively [71]. In addition, the rotational frequency of head rotation is limited to 2.5 Hz for yaw and roll axes, and 3.6 Hz for the pitch axis [72]. Therefore, a rotary stage capable of simulating head rotation around the rotational axes was designed, as shown in Fig. 6a. The rotary stage is composed of three servomotors controlled by an Arduino UNO microcontroller with code written in Arduino IDE language [33]. The stage has been programmed to oscillate sinusoidally, with a soft start and stop to minimize transients in the measurements and stresses on the stage and device under investigation. Measurements are also only recorded after five cycles, to ensure that the system has reached a steady state. The VGN/PDMS flow sensor was equipped with the ultra-thin copper wire (0.012 mm diameter) and carefully embedded into the ampulla via a hole at the bottom of LSCC. As previously mentioned, the base of the sensor has been made from 10:1 PDMS mixture, so a thick layer of double-sided tape was used for sealing the gap between the sensor base and the LSCC, following which the LSCC was filled with the deionized water (density and viscosity of 1 g mL^−3^ and 1 cP, respectively). Finally, the whole system was positioned at the center of the rotary stage, as shown in Fig. 6a.

By applying a specific frequency and rotational angle to the rotary table around a particular rotational axis, fluid flow is generated inside the LSCC, which deforms the flow sensor. The resultant deformation in the VGNs thin film varies the electrical resistance of the flow sensor, which is translated to a voltage via a Wheatstone bridge circuit. To avoid wire vibration and potential breakage, the circuit was attached to the base of the stage rotating, as indicated in Fig. 6a. The voltage obtained was filtered through the SRS560 low-noise preamplifier with unity gain, and the voltage was measured by the DAQ device. Finally, the results were recorded and displayed with NI LabVIEW Signal Express software to evaluate the sensor output.

To demonstrate the performance of the newly fabricated flow sensor for human balance, the VGNs/PDMS flow sensor was used to mimic the sensory hair cells inside LSCC by capturing oscillatory fluid flow due to rotation of the stage. The sensor response to the rotary stage for a broad range of frequencies and amplitude (rotational angles) was investigated thoroughly. In Fig. 6b–e, the stage and the sensor outputs were plotted in blue and red, respectively, for 60-degree amplitude and four different frequencies of yaw axis rotation. The plots show the phase difference between the sensor output and the stage position. Clearly, fluid inertia inside LSCC in each cycle causes a significant and fixed delay between the stage rotation and fluid flow. The flow sensor was able to detect the change of the velocity and direction of the fluid flow. This was shown by the existence of two peak signal values at the output voltage and the approximately constant discrepancy between these two peaks during each cycle. By comparing the sensor output at different frequencies, it can be seen that the small peak emerges at a low frequency (0.5 Hz) and grows with increasing frequency. The justification for the phenomenon is the presence of uneven hydrodynamic forces acting on either side of the flow sensor, due to the asymmetric geometry of the LSCC. Moreover, with the increasing speed of the stage rotation, the flow sensor shows a corresponding and significant response. In our previous project [33], a different flow sensor embedded in the LSCC was tested and also observed each of these behaviors.

**Fig. 7:**
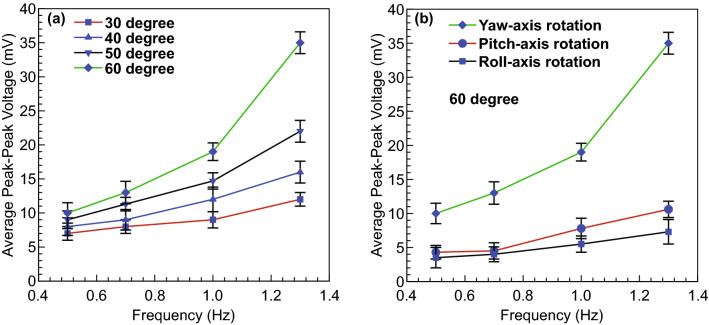
**a** Sensor response to various rotational angles and frequencies in yaw axis rotation and **b** changes in rotational axes

The performance of the flow sensor with different physiological conditions and different rotational axes is examined in Fig. 7. The obtained results indicate that for the case of yaw-axis rotation, either increasing the rotational angle or frequency results in an increase in average peak–peak output voltage (Fig. 7a). The greater augmentation is observable at greater frequency and amplitude of rotation due to increase in fluid momentum and the secondary flow inside the LSCC, leading to larger relative fluid velocity at the sensor, causing increased deformation of the sensor. For instance, the sensor output in terms of average peak–peak voltage for 30-degree and 60-degree amplitudes from 0.5 to 1.3 Hz rises to roughly twofold and fourfold, respectively. Figure 7b presents the results obtained from changing the rotational axis for 60-degree amplitude and varying the frequency. As expected, the sensor output for the case of LSCC rotation around the yaw axis is significantly higher than that of around roll and pitch axes. This phenomenon arises because the LSCC is aligned in the same rotation plane as the yaw axis rotation, so the fluid inside the LSCC will have a stronger induced flow, compared to the cases of rotation around roll and pitch axes.

**Fig. 8:**
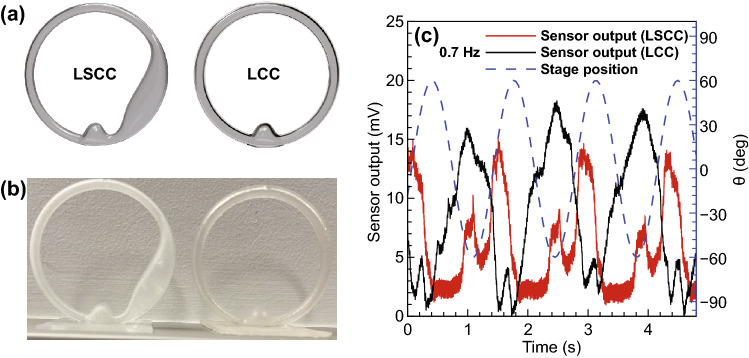
**a** Modeling of LSCC and LCC, **b** 3D printed LSCC and LCC, and **c** sensor response to stage rotation for two cases of LSCC and LCC

To evaluate the effectiveness of the VGN/PDMS flow sensor with changes of the shape of the canal, two canals were designed with different geometries, one with an utricle (semicircular canal) and the other without the utricle (circular canal), as presented in Fig. 8a, b. In both canals, the flow sensor was embedded into the ampulla. The sensor output for the LSCC and the lateral circular canal (LCC), and the stage position are presented in Fig. 8c for 60-degree amplitude and 0.7 Hz frequency. The results demonstrate that for the LCC, there is only a single peak, which occurs simultaneously with the smaller peak of the LSCC. Due to the symmetric geometry of the LCC, the amount of bending of the VGNs thin film in the middle of the cycle is equal to that of the opposing half of the cycle, so the sensor responds to fluid flow with one peak. For the LSCC, secondary flow and unequal pressure on either side of the sensor lead to the VGNs thin film unevenly bending, so two peaks are observable.

## Conclusions

In this work, a newly developed flow sensor with ultra-high sensitivity and performance has been designed using PDMS composites with vertical graphene nanosheets (VGNs). The VGNs/PDMS thin film sensor was subjected to oscillatory stimuli in contact with the sensor, oscillatory flow stimuli, and steady-state flow to investigate its flow sensing capability. The sensitivity of the sensor was measured using steady-state flow tests to be 103.91 mV (mm/s)^−1^, which is far higher than that of other flow sensors. The obtained threshold velocity for detecting steady-state flow in a straight channel is 1.127 mm s^−1^. This flow sensor demonstrates an upward trend on the oscillatory flow with changing frequency and amplitude, while the sensor has an extremum (1.1 Hz) in the frequency sweep of oscillatory stimuli in contact with the sensor. The general principle of flow sensors is based on piezoresistivity of the sensor in response to the bending deformation.

A significant application of this innovative flow sensor is to detect oscillatory flow inside the semicircular canal by mimicking the behavior of the sensory hair cells in the ampulla and using this for detecting head movement. SLA 3D printing technology was used for fabrication of the bioinspired lateral semicircular canal (LSCC) so that the flow sensor embedded inside the LSCC could be tested on the rotary stage. The sensor was evaluated at different frequencies and rotational angles in three separate rotational axes, namely yaw, pitch, and roll. The obtained results indicate high sensitivity and excellent performance of the sensor across a wide range of frequencies and amplitudes. The sensor could detect two peaks in a single cycle, as a result of the asymmetric geometry of LSCC and a phase difference due to fluid inertia. Because of the existence of an open path to the sensor in rotation around yaw axis, the sensor showed higher sensitivity than that of around pitch and roll axes, which is consistent with the physiology of the vestibular system. The flow sensor was also used to test the variation of the geometry of the semicircular canal by omitting the utricle in the LSCC, and observing that only one peak was present, and occurred at a similar time in the cycle to the smaller peak in the LSCC.

This work puts forward a newly fabricated graphene-based flow sensor with excellent features, ultra-high sensitivity, linearity, and low-velocity detection, offering a viable alternative for mimicking sensory hair cells in the vestibular system and hearing system.

## Electronic supplementary material

Below is the link to the electronic supplementary material.

Supplementary material 1 (PDF 594 kb)
