# Supplementary material for: Development of an Ultra-Sensitive and Flexible Piezoresistive Flow Sensor Using Vertical Graphene Nanosheets
Source: Nanomicro Lett. 2020 May 11;12:109. doi: 10.1007/s40820-020-00446-w (PMC7770822; doi:10.1007/s40820-020-00446-w)
Supplement: Supplementary file 1 — Supplementary material 1 (PDF 594 kb) [file 40820_2020_446_MOESM1_ESM.pdf]

Supporting Information for

Development of an Ultrasensitive and Flexible Piezoresistive Flow Sensor Using Vertical Graphene Nanosheets

Sajad Abolpour Moshizi[1], Shohreh Azadi[1], Andrew Belford[1], Amir Razmjou[2], Shuying Wu[1], Zhao Jun Han[3], Mohsen Asadnia[1,*]

[1]School of Engineering, Macquarie University, Sydney, NSW 2109, Australia

[2]UNESCO Centre for Membrane Science and Technology, School of Chemical Science and Engineering, University of New South Wales, Sydney, NSW 2052, Australia

[3]CSIRO Manufacturing, PO Box 218, 36 Bradfield Road, Lindfield, NSW 2070, Australia

*Corresponding author. E-mail: mohsen.asadnia@mq.edu.au (Mohsen Asadnia)

S1 Biology of Semicircular Canal

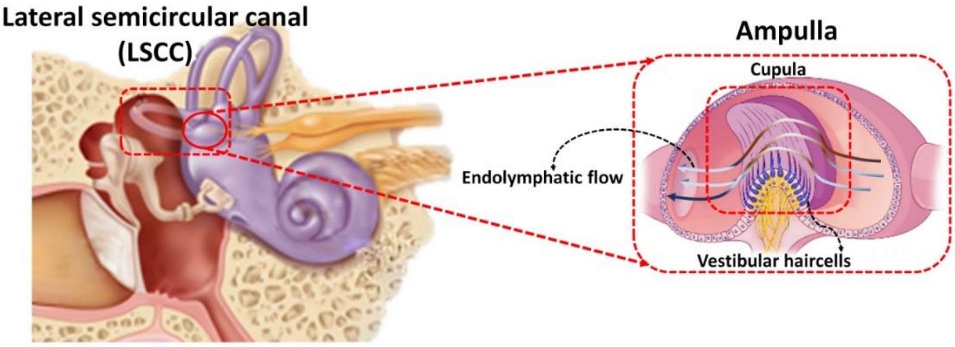

Fig. S1 Schematic of the biological inner ear and semicircular canal

S2 Rheological Properties of Poly(dimethylsiloxane)

The rheological properties of different mixtures of PDMS including the prepolymer and curing agent (Sylgard 184, Dow Corning Australia) with weight ratios of 10:1, 15:1, 20:1, 25:1 and 30:1 were analysed using a stress-controlled shear rheometer (MCR 302, Anton Paar, Graz, Austria) with a parallel plate. Each mixture was cut into a disk with a diameter of 20 mm and a thickness of 2 mm. By measuring storage modulus (G') and loss modulus (G") for a frequency sweep over a range of 0.01–50 Hz under a constant strain of 1%, the amplitude-linear viscoelastic region has been obtained, as can be seen in Fig. S2a. Moreover, all samples were prepared as a disk shape for amplitude sweep experiments with a constant angular velocity of 10 rad/s under the shear strain range of 0.01% - 100%, as presented in Fig. S2b.

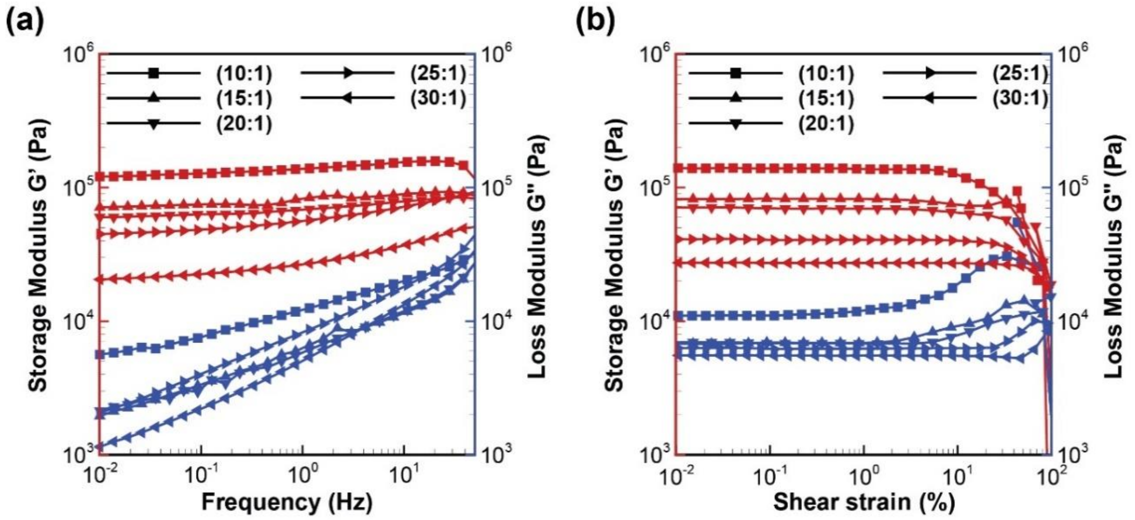

Fig. S2 Rheological properties of several mixtures of PDMS, storage modulus G' (Pa) and loss modulus G" (Pa) versus, **a** frequency sweep and **b** shear strain sweep

The mechanical properties of these mixtures of PDMS were tested with a universal testing machine (MTS Exceed E42, MTS Systems Corporation, USA) and studied through a stress-strain curve, as presented in Fig. S3. The samples were prepared in the form of strips with a dimension of 40 mm (length) × 7 mm (width) × 2 mm (thickness). For all tests, the crosshead speed was set to 5 mm/min until the sample was damaged. The slope of the stress-strain curve represents Young's modulus for each sample. The results obtained indicate that Young's modulus, loss modulus and storage modulus strongly depends on the weight ratio of the PDMS components, and the studied parameters have a downward trend with increasing the weight ratios.

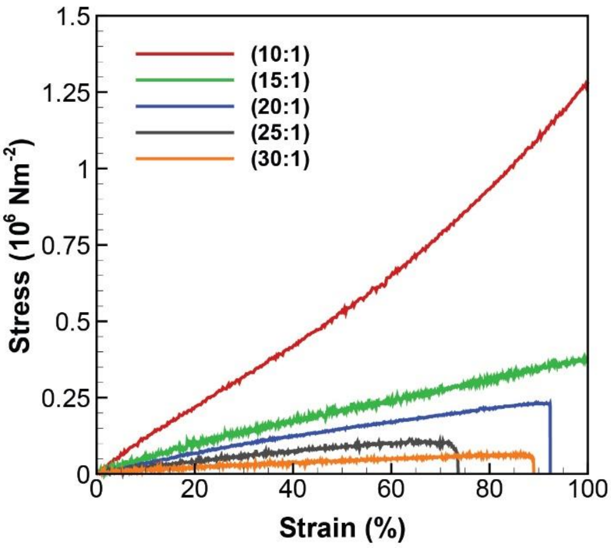

Fig. S3 PDMS mechanical properties, stress versus strain

S3 Bias Stability Experiment

The bias stability of the flow sensor represents the noise characterization of the sensor under no external force. We used a voltage divider circuit (Fig. S4) connecting a NI DAQ device to measure the resistance of the sensor.

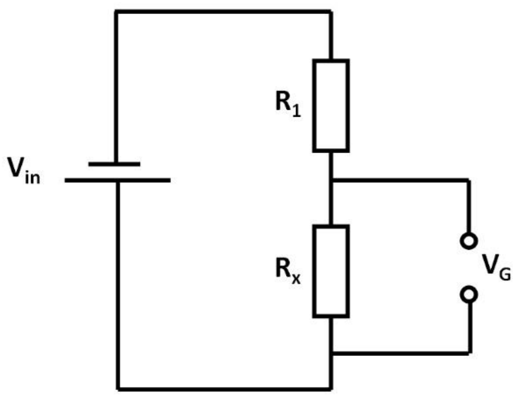

Fig. S4 Voltage divider circuit

R_x is the resistance of the sensor, V_G is measured by a NI DAQ device, V_{in} (6.366 V) is the supply power, and R_1 (=9.96 kΩ) is the auxiliary resistance. Then by using Eq. S1, the resistance of the sensor (R_x) is obtainable as below:

$$R_x = \frac{V_G R_1}{V_{in} - V_G} \qquad \text{(S1)}$$

The results show that the resistance of the sensor has 10Ω deviation from the baseline, as presented in Fig. S5.

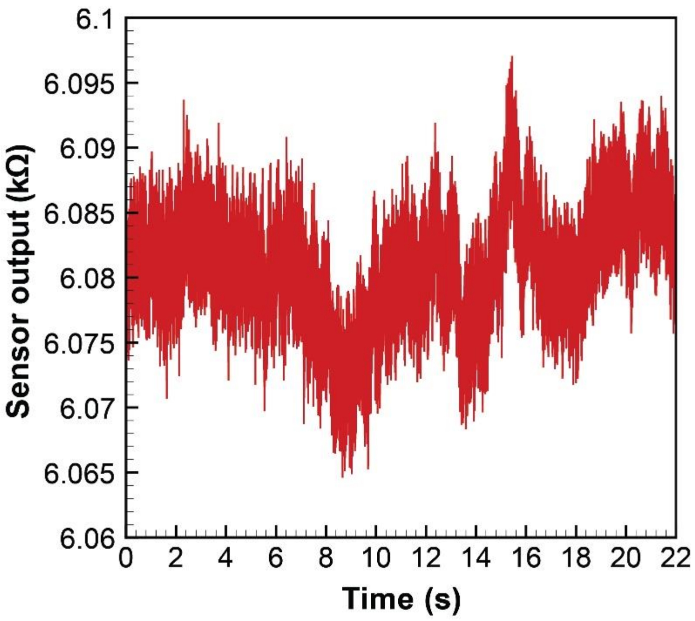

Fig. S5 Bias stability of the VGNs/PDMS flow sensor over time

S4 Sensor Output in Terms of Voltage and Resistance

We used a voltage divider circuit to measure the resistance changes of the sensor when the sensor was exposed to the fluid flow. Fig. S6 shows the sensor response to the applied flow with a velocity of 30 mL min^{-1} in terms of voltage (mV) and resistance (kΩ).

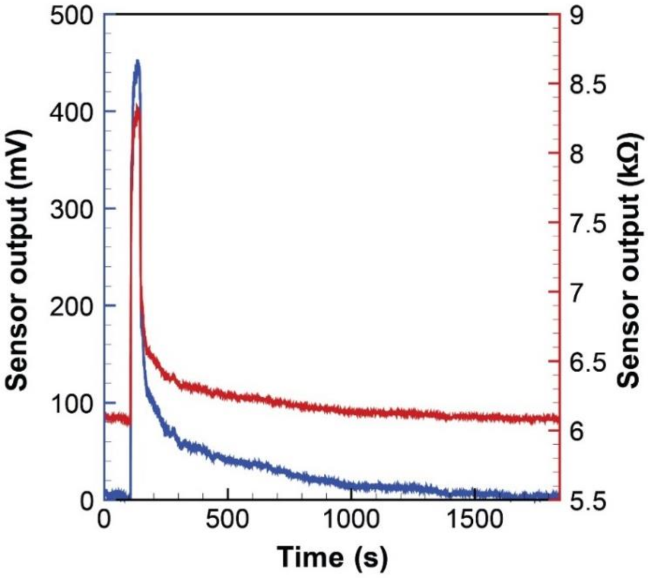

Fig. S6 Steady-state flow testing inside a straight channel, sensor output in terms of voltage (mV) and resistance (kΩ) at 30 mL min^{-1}

S5 Sensor Flow Response in terms of Resistance

The sensor response in terms of the sensor resistance as a function of flow velocity is plotted in Fig. S7. This measurement was conducted by the voltage divider circuit connecting to the NI DAQ device

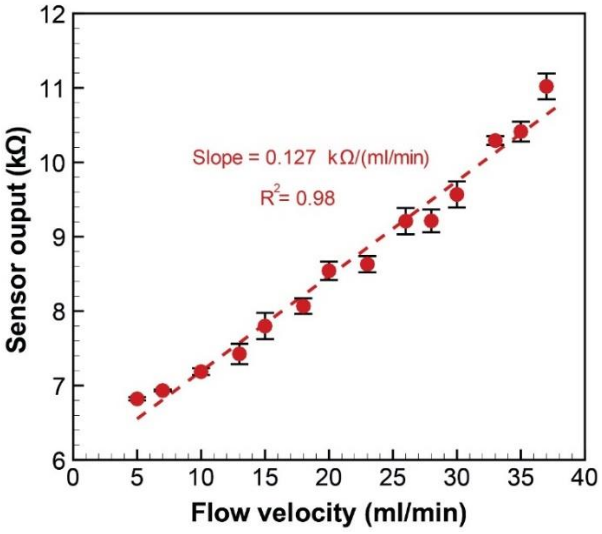

Fig. S7 Sensor output in terms of sensor resistance as a function of flow velocity (calibration plot)

S6 Magnetic Resonance Imaging (MRI) Results

To find the morphology of the inner ear, a healthy human underwent a 3T magnetic resonance imaging (MRI) scanner (Siemens, Verio). The acquisition parameters were echo time (TE) = 93 ms, repetition time (TR) = 9700 ms, field of view (FOV) = 197×220 mm, and flip angle =128°.The inner ears in either side of the human head were recognized by examining among several layers. In Fig. S8, we can see three interconnected semicircular

canals and the cochlea. After finding the components of the lateral semicircular canal, we measured the diameters of the utricle (d2), slender duct (d3), and ampulla (d1), as well as the duct curvature (2R).

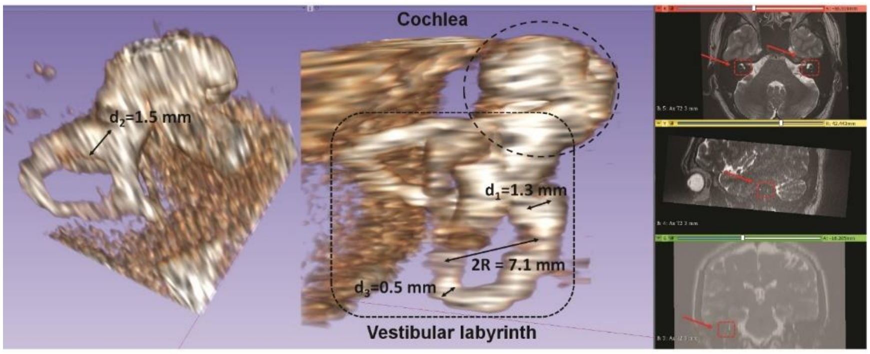

Fig. S8 Volume rendering from MRI images of a human head and finding the inner ear including the cochlea and vestibular labyrinth
